# The moderating role of self-efficacy in the relationship between occupational stress and mental health issues among nurses

**DOI:** 10.1038/s41598-024-66357-7

**Published:** 2024-07-10

**Authors:** Sima Rafiei, Saber Souri, Zahra Nejatifar, Mohammad Amerzadeh

**Affiliations:** 1 Social Determinants of Health Research Center, Research Institute for Prevention of Non-Communicable Diseases, Qazvin University of Medical Sciences, Qazvin, Iran; 2 Student Research Committee, School of Public Health, Qazvin University of Medical Sciences, Qazvin, Iran

**Keywords:** Occupational stress, Mental health, Self-efficacy, Nurses, Psychology, Health care

## Abstract

Occupational stress is a complex concept resulting from interaction among personnel, work environment, and cultural contexts. It can cause mental health issues, including clinical mental disorders, as well as emotional challenges like depression, anxiety, cognitive difficulties, and feeling sad. As a vulnerable group, healthcare professionals, especially nurses, experience a high rate of occupational stress. Therefore, we aimed to study the relationship between occupational stress, mental health problems, and self-efficacy among the nursing population. A cross-sectional study was conducted among 365 nurses selected through a two-stage sampling process from tertiary hospitals in Qazvin, Iran, between July and September 2022. Study variables included occupational stress measured by the persian version of the health and safety executive management standards revised indicator tool (MS-RIT), the general health questionnaire containing 28 questions measuring psychological distress in four primary areas of somatic and anxiety symptoms, sleep disorders, social dysfunction, and depression [GHQ–28], and general self-efficacy [GSE–10]. The mentioned variables and some of the nurses’ demographic characteristics, including age, gender, education, and work experience, were analyzed using moderated multiple regression, descriptive statistics, and the Pearson correlation coefficient.The Pearson correlation analysis revealed a statistically significant association between self-efficacy and occupational stress (r = 0.62, P = 0.000) and self-efficacy and mental health (r = 0.67, P = 0.01). Regression analysis demonstrated that occupational stress accounted for 42% of the variation in mental health (R^2^ = 0.42, P < 0.01). The results also showed that self-efficacy moderates the relationship between occupational stress and mental health, with a significant effect (P < 0.01). The elevated prevalence of occupational stress and the concerning state of mental health among nurses highlight the need for the implementation of intervention programs, including stress prevention strategies at the workplace through organizing better working shifts, as well as increasing nurses’ self-efficacy and their effective participation in work-related tasks aiming to enhance working conditions for nurses.

## Introduction

Occupational stress is a multifaceted phenomenon that arises from the interplay among personnel, the work environment, and cultural contexts^1,2^. It manifests as a pattern of reactions experienced by employees when they confront work demands that exceed their knowledge and abilities, thus challenging their capacity to cope^3^. This type of stress causes destructive physical, mental, and emotional reactions in staff due to an imbalance between job demands and personal time, skills, abilities, or resources^4^. The consequences of occupational stress are far-reaching, impacting the well-being of personnel, including their health, family life, job satisfaction, and performance. Organizations are also affected, experiencing increased intentions to leave among employees, higher staff turnover rates, and subsequent expenditures to enhance personnel morale^5–7^.

Healthcare professionals, particularly nurses, are considered a vulnerable work group with a heightened susceptibility to occupational stress. Various work-related stressors contribute to this situation, such as heavy workloads, improper shift work, lack of social support, potential conflicts with physicians, and the challenging nature of critical conditions, patients, and their families^8–10^. Such workplace stressors and subsequent mental health conditions might cause adverse effects on nurses and their performance, ultimately impacting patient outcomes^11^.

Interventions to reduce occupational stress primarily focus on implementing changes in organizational policies and individual procedures^12^. Some studies emphasized the importance of work resources in promoting individuals’ psychological capacities by providing flexible and adaptable working conditions^13^. Condensed work hours and set flexible working days are among the critical policies to benefit nurses and enhance their work-life balance, reduce their stress and burnout, raise productivity, and consequently improve the quality of patient care provision^14^. However, among individual factors, self-efficacy is crucial in mitigating the effects of occupational stress, particularly for nurses who frequently encounter workplace stressors. Self-efficacy refers to an individual's belief in their ability or confidence to cope effectively with stressful events^15^. Providing regular training and practical courses to empower nurses in handling challenging situations, establishing a mental consultation center in a hospital to assess nurses’ psychological needs periodically, offering them feedback, and giving them mentors to help improve their knowledge, skills, and mindfulness are among key organizational policies and procedures that emphasize the role of personal resources of staff, such as self-efficacy, in reducing the negative impact of job stress^16–19^. A meta-analysis indicated that higher levels of self-efficacy are associated with lower rates of distress and symptoms of post-traumatic stress disorder (PTSD)^20^. Researchers also found that self-efficacy can reduce immediate and long-term stress levels across contexts^21^. In line with these findings, evidence supported the idea that work-related stress can decrease self-efficacy, resulting in anxiety, depression, and other negative emotions. Likewise, Mahdizadeh et al. reported a negative correlation between occupational stress and self-efficacy among nurses^22^. Accordingly, they highlighted the need to improve the knowledge of clinical staff on how self-efficacy affects occupational stress, helps staff address challenging situations, improves their caregiving skills, and avoids their mental health issues^22,23^.

Work-related stressors significantly impact employees’ beliefs, health, and performance. However, only a few studies have explored the relationship between occupational stress and self-efficacy, specifically among nurses. Therefore, our study aimed to investigate the relationship between occupational stress, mental health problems, and self-efficacy within the nursing population while also examining the mediating effect of self-efficacy in the relationship between occupational stress and mental health problems.

## Methods

### Study design

This study employed a cross-sectional, analytical design and targeted nurses at training hospitals affiliated with the Qazvin University of Medical Sciences. The data collection took place between July and September 2022.

### Study participants

The study focused on nurses in different clinical departments across four training hospitals in Qazvin, Iran. Using the Cochrane formula, the sample size was estimated at 365. We applied a two-stage sampling method so that in the first stage, we selected the hospitals randomly and used the proportional allocation method to select an appropriate number of participants from each hospital. To be eligible, nurses had to work more than one year in their current job and had no history of mental disorders.

### Data collection

The data collection process involved using three questionnaires: (1) occupational stress, (2) self-efficacy, and (3) mental health. The initial section of the questionnaire focused on the demographic and job-related characteristics of the participating nurses. These characteristics encompass age, gender, educational background, and work experience.

The Health & Safety Executive (HSE) Management Standards Revised Indicator Tool (MS–RIT) was used to assess psychosocial risks contributing to workplace stress among nurses using 35 self-report questions^24^. The questions were organized into seven main dimensions, including job demand (8 items related to work procedures, workload, and work environment), control (6 items about the degree to which a person has control over the work), managerial support (5 items evaluating support and resources provided by the employer, organization and line management), peer support (4 items concerning support from colleagues), communication (4 items addressing potential conflicts at work and making positive working atmosphere to deal with unacceptable behavior), job role (5 items around understanding job roles and avoiding conflict roles), and change with three items about how organizational change is managed^24,25^. Response to each question could be given in a 5-point Likert-type scale (‘always’ to ‘never’ and ‘strongly disagree’ to ‘strongly agree’), with higher scores representing lower levels of occupational stress. The validity and reliability of the questionnaire had been tested and approved in previous studies like the research conducted by Marzabadi and Gholami Fesharaki^25^. The validity and reliability of the Persian version of the questionnaire were 78 and 65%, relatively confirming MS-RIT as a valid and reliable questionnaire for investigating occupational stress^24–26^.

Mental health was the second study variable evaluated by the general health questionnaire (GHQ–28) developed by Goldberg in 1979^27^. This scale measures mental health in four areas: anxiety, sleep disorders, somatic symptoms, and social dysfunction, each encompassing seven items scoring on a 4-point Likert scale (ranging from ‘never true’ = 0 to ‘always true’ = 3). A score between 10 and 12 is considered typical, above 15 shows distress, and scores above 20 represent severe psychological distress. In a study by Nourbala et al., the reliability and validity of the standardized Persian version of GHQ-28 were relatively reported to be 84.7 and 93.8%^28^.

Finally, a 10-item general self-efficacy (GSE–10) scale was used to assess self-efficacy among study participants based on a 4-point Likert scale (ranging from ‘totally incorrect’ = 10 to ‘totally correct’ = 40). Higher scores on this tool showed higher levels of self-efficacy and coping ability of staff in dealing with daily challenging conditions and adaptation to different kinds of stressful life events. The validity of the developed scale by Nezami et al. was 0.93, indicating that the questionnaire measures what it is intended to measure^29^.

### Data collection process

Before starting the data collection process, we gained ethical approval from the University Ethics Committee to ensure compliance with the ethical principles in all study phases, from recruitment to data collection, data analysis, and data storage. These principles mainly included anonymity of data, data protection, data privacy, respect for the dignity and autonomy of study participants, their beneficence, informed consent, and avoidance of conflict of interest. Afterward, the researcher who had been trained to gather information, referred to study hospitals, explained the study objectives to nurses, controlled the inclusion criteria, and obtained informed consent. Then, the researcher provided the questionnaires to study samples and asked them to complete them in two weeks. After a week from the time of distributing questionnaires, a reminder email was sent to nurses. Then, at the end of the deadline, the researcher collected completed questionnaires by visiting the hospitals and affiliated care units.

### Data analysis

To examine the study hypothesis, moderated multiple regression analysis was employed in STATA v. 13.1 to explore the impact of self-efficacy on the association between occupational stress and the mental health condition of nurses. In addition, descriptive statistics and the Pearson correlation coefficient were utilized to analyze the data.

### Ethics approval and consent to participate

The Ethical Committee of the QUMS- Iran approved this study. Ethical code: IR.QUMS.REC.1401.079 and all participants signed a written informed consent form before participation. The Ethical Committee approved all experimental protocols. All methods were carried out in accordance with relevant guidelines and regulations. We provided the participants or their legal guardian(s) with an information sheet, reassured them about anonymity, freedom to withdraw, confidentiality and explained the study’s purpose, and obtained their informed consent form.”

## Results

Out of the 365 questionnaires distributed to nurses, 320 questionnaires were returned, resulting in a response rate of 87.67%. The majority of the participants in the study were female, accounting for 88% of the sample. The age range of the participants fell between 35 and 45 years old. Regarding educational qualifications, approximately 73% of the respondents held a BSc degree. Regarding work experience within the current hospital department, nearly 69% of the participants had one to five years of experience. As findings in Table 1 depict, a statistically significant relationship was found between age, work experience, and occupational stress among study participants (P < 0.05).

**Table 1:** The status of occupational stress among nurses.

Characteristics		Frequency	%Frequency	P-value
Gender	Male	39	12	0.57
	Female	281	88	
Marital status	Married	230	72	0.24
	Single	100	28	
Educational level	BSc	233	73	0.19
	MSc	57	18	
	PhD	30	9	
Working experience	< 5	220	69	0.04
	5–9	83	25.9	
	≥ 10	17	5.1	

	Mean	SD	–	P-value
Age	40	10	–	0.03

The mean (SD) score for occupational stress among the nurses was 94.2 (37.8) between 35 and 175, indicating moderate stress among nurses. Regarding mental health status, the mean (SD) score was estimated at 8.3 (10.3) within a range of 3 to 61, indicating a relatively undesirable mental health status among study participants. Finally, the mean (SD) score for general self-efficacy was 31.4 (13.2) within a range of 10 to 40, suggesting a moderately high level of self-efficacy among nurses and representing a high level of confidence in their ability to deal with challenging situations.

The results of Pearson correlation analysis indicated a significant relationship between self-efficacy and occupational stress (r = 0.62; P < 0.01), self-efficacy and mental health (r = 0.67; P < 0.05), occupational stress and mental health (r = −0.412; P = 0.00) among participants. These findings suggest that self-efficacy and occupational stress are associated with mental health outcomes among the nursing population. Table 2 provides detailed information on the correlation analysis results.

**Table 2:** Correlations between study variables.

Scale/subscale	Stress	Demand	Control	Managerial support	Peer support	Communication	Role	Change	Self-efficacy	Mental health
Stress	–								0.62 (0.000)	0.72 (0.02)
Demand	0.422 (0.001)	–								
Control	0.523 (0.01)	0.312 (0.00)	–							
Managerial support	0.579 (0.00)	0.367 (0.02)	0.429 (0.00)	–						
Peer support	0.598 (0.010	0.266 (0.00)	0.511 (0.00)	0.562 (0.00)	–					
Communication	0.452 (0.00)	0.472 (0.00)	0.365 (0.00)	0.294 (0.01)	0.422 (0.00)	–				
Role	0.426 (0.00)	0.212 (0.00)	0.226 (0.01)	0.375 (0.00)	0.382 (0.00)	0.366 (0.00)	–			
Change	0.649 (0.00)	0.235 (0.01)	0.576 (0.00)	0.446 (0.00)	0.362 (0.00)	0.347 (0.00)	0.252 (0.01)	–		
Self-efficacy	0.62 (0.000)	0.301 (0.00)	0.427 (0.00)	0.459 (0.00)	0.397 (0.00)	0.322 (0.02)	0.142 (0.01)	0.115 (0.01)	–	
Mental health	0.72 (0.02)	−0.225 (0.01)	−0.289 (0.01)	−0.319 (0.00)	−0.472 (0.00)	−0.465 (0.00)	−0.232 (0.01)	−0.169 (0.01)	−0.412 (0.00)	–

Regression analysis was performed to examine the relationship between occupational stress and mental health. The results, as presented in Table 3, showed that the independent variable (occupational stress) accounted for 42% of the variation in the dependent variable (mental health) (R^2^ = 0.42, P < 0.01). This indicates that occupational stress significantly influences mental health outcomes among nurses.

**Table 3:** Regression analysis for the relationship between occupational stress and mental health.

Model	R	R^2^	Adjusted R^2^	Standard error of the estimate	Change statistics				
					Change in R^2^	F change	df1	df2	P-value
1	0.68	0.42	0.41	5.7	0.28	62.7	1	112	0.001

Then, we considered whether occupational stress might positively predict self-efficacy and whether self-efficacy significantly affects mental health. To test the study hypothesis, a moderated multiple regression analysis was conducted, and the moderating effect of self-efficacy on the relationship between occupational stress and mental health was tested. Findings supported the hypothesis and demonstrated that occupational stress had a substantial positive impact on self-efficacy (*β* = 0.26, P < 0.01), and self-efficacy negatively predicted mental health (*β* = −0.46, P < 0.01) in nurses. Therefore, the hypothesis of the moderating role of self-efficacy in the relationship between occupational stress and mental health was confirmed (P < 0.01). As age and work experience were found to have a statistically significant relationship with occupational stress, these two variables were also included in the model (Table 4).

**Table 4:** The mediating role of self-efficacy in the relationship between occupational stress and mental health.

Regression model		Model fit		Standardized coefficients (β)	Unstandardized regression coefficients (B)	t	P-value
		R	R^2^				
Self-efficacy	Age	0.38	0.26	0.02	0.04	5.12	< 0.01
	Work experience			0.34	0.63	2.18	
	Occupational stress			0.26	0.30	2.75	
	Mental health			−0.25	−1.03	−3.42	
Mental health	Age			0.52	1.65	1.34	< 0.01
	Work experience	0.52	0.46	0.04	0.209	0.45	
	Self-efficacy			−0.46	−2.57	−3.36	
	Occupational stress			0.17	0.32	4.25	

## Discussion

This study aimed to examine the relationship between occupational stress, mental health problems, and self-efficacy among nurses and explore the potential mediating effect of self-efficacy on the occupational stress-mental health relationship. Our findings revealed a high prevalence of occupational stress and a low level of mental health status among the nursing population, indicating the challenging nature of nursing work and emphasizing the need for interventions to improve their working conditions.

Consistent with previous research^30,31^, our study found a high rate of occupational stress among nurses. These findings provide valuable insights for policymakers and healthcare managers, prompting them to develop effective preventive interventions to manage occupational stress. Such interventions may include workplace interventions aimed at improving job demands, enhancing control over work conditions, providing adequate personnel support, fostering positive relationships, and implementing organizational changes^32^.

By addressing the occupational stress experienced by nurses, healthcare organizations can create a healthier and more supportive work environment, which can positively impact mental health and nurses’ well-being. These findings highlight the significance of implementing targeted interventions and policies to address occupational stress and promote mental health among nurses, ultimately improving their job satisfaction and performance.

Our findings align with previous research^33–35^, which has consistently reported high levels of mental health problems among nurses. These findings indicate that nurses are at risk of experiencing mental health issues. The results underscore the urgent need for interventions aimed at improving the mental health status of nurses, with particular attention to addressing somatic and anxiety symptoms, sleep disorders, and social dysfunction.

A study conducted in Spain involving 1777 nurses found a significant association between self-efficacy and stress levels^36^. Our results indicated that higher self-efficacy scores were correlated with lower stress scores among nurses. These findings align with previous research^37–39^, which also demonstrated that lower self-efficacy was linked to higher stress levels. Nurses who perceived themselves as more effective in challenging situations reported lower stress, fear, or anxiety levels. This suggests that nurses with higher self-efficacy beliefs felt more confident in their ability to manage and cope with stressful conditions successfully.

These studies collectively support the job demands-resources model, which posits that the personal resources of employees, such as emotional intelligence and self-efficacy, play a crucial role in mitigating the negative impact of job demands and enhancing job performance^40^. By having higher levels of self-efficacy, nurses are better equipped to handle the demands and challenges they face in their work environment, reducing the adverse effects on their well-being and job performance.

Our findings demonstrated that self-efficacy moderated the association between occupational stress and nurses' mental health. However, another study conducted in Iran reported contrasting results, indicating that self-efficacy did not moderate the relationship between occupational stress and mental health problems^32^. These divergent outcomes could be attributed to variations in employee characteristics and the specific contexts in which the studies were conducted, including differences in patient populations and cultural factors. Nonetheless, another study, consistent with our findings, supported that employees with high levels of self-efficacy exhibit a heightened sense of control, enabling them to moderate the relationship between stress and health outcomes^41^.

Our study findings established a significant association between occupational stress and mental health problems among nurses. Specifically, we observed that all domains of occupational stress hurt nurses' mental health, which is consistent with previous research^6,42^. A study conducted in Iran also reported a significant relationship between occupational stress and mental health, demonstrating that job stress and mental disorders co-occurred^31^. Additionally, another study highlighted the detrimental effects of occupational stress on job satisfaction and nurses' mental health problems. This study underscored the role of poor human resources management, inadequate resources, and occupational risks in generating occupational stress and subsequent mental health issues^42^.

The impact of poor human resources management on staff morale, leading to depersonalization, job dissatisfaction, and mental health problems, was evident in the literature^44^. Moreover, insufficient resources contributed to feelings of insecurity regarding the acquisition and maintenance of essential resources, thereby triggering occupational stress and potential mental health problems^42^.

These findings collectively emphasize the significance of addressing occupational stress and its underlying causes in healthcare organizations. By improving human resources management, ensuring adequate resources, and managing occupational risks, healthcare institutions can promote better mental health outcomes among nurses. Such interventions are essential for fostering a positive work environment, enhancing job satisfaction, and mitigating the adverse effects of occupational stress on mental health.

Several studies have explored interventions to enhance nurses' performance by improving their working conditions. These interventions encompass training courses focusing on managing occupational stress and empowering assertiveness and relaxation techniques. By providing nurses with these necessary skills, as well as adequate resilience, self-efficacy, and active job engagement, they can effectively handle stressors in the workplace, which consequently would lead to the provision of quality services to patients and safety care with the minimum occurrence of adverse events^45^. Thus, managers and healthcare administrators need to provide adequate opportunities for training and skill development of nurses to improve their resilience, self-efficacy, and job engagement. This might also include a series of strategies that promote the staff’s coping abilities and increase their psychosomatic well-being through offering interpersonal communication skills, mentoring programs, emotional support, and opportunities for outdoor activities.

Our study has several limitations. First, the generalizability of our findings may be limited because the study was conducted in only one province. The results may be representative of only some of the nursing population in Iran or other regions. Therefore, it would be valuable to replicate this study in multiple provinces, different hospital sectors, and private hospitals to compare the findings and enhance the generalizability of the results.

Second, the study design was cross-sectional, which might have limited the ability to establish causal relationships between variables. As cross-sectional studies provide a snapshot of data at a specific time, it is difficult to determine the temporal sequence of events or the direction of the relationships observed. Third, the study sample might not be an appropriate representative of all nurses in Iran, so it is essential to expand the research to a national level and confirm the accuracy and reliability of the findings. Mixed-method studies that combine qualitative methods with quantitative surveys are suggested to be applied in future research studies to obtain a deeper understanding of the factors influencing nurses’ occupational stress and mental health status. Future research could also employ longitudinal designs to better understand the dynamic nature of the variables under investigation and strengthen causal inferences.

## Conclusion

The elevated prevalence of occupational stress and the concerning state of mental health among nurses underscore the need for implementing additional intervention programs to enhance their working conditions. Our study findings support the notion that occupational stress significantly impacts nurses’ mental health while highlighting the moderating role of self-efficacy in this relationship. Further research is warranted to explore strategies to manage occupational stress and improve nurses’ mental health effectively. Such research endeavors can contribute to mitigating the adverse effects of occupational stress on nurses' mental well-being. Achieving this objective requires implementing evidence-based policies that foster better working conditions for nurses, ensuring they feel secure and enabling them to perform their duties more effectively. By creating a supportive and conducive work environment, healthcare organizations can play a vital role in promoting nurses' mental health and overall well-being. These interventions should be guided by rigorous research and tailored to address nurses' specific needs and challenges in their occupational settings.

## Data Availability

The datasets used and/or analyzed during the current study available from the corresponding author on reasonable request. The entire dataset is in Farsi language. The Data can be available in English language for the readers and make available from the corresponding author on reasonable request.
